# Supplementary material for: An investigation of the immune epitopes of adeno-associated virus capsid-derived peptides among hemophilia patients
Source: Mol Ther Methods Clin Dev. 2024 Apr 2;32(2):101245. doi: 10.1016/j.omtm.2024.101245 (PMC11039395; doi:10.1016/j.omtm.2024.101245)
Supplement: Document S1. Figures S1–S4 and Table S1 [file mmc1.pdf]

OMTM, Volume *32*

## Supplemental information

# An investigation of the immune epitopes of adeno-associated virus capsid-derived peptides among hemophilia patients

**Li Liu, Bingqi Xu, Lingling Chen, Jia Liu, Wei Liu, Feng Xue, Sizhou Feng, Erlie Jiang, Mingzhe Han, Wenwei Shao, Lei Zhang, and Xiaolei Pei**

MAADGYLPDWLEDTLSEGIRQWWKLKPGPPPPKPAERHKDDSRGLVLPGYKYLGPFNGL
DKGEPVNEADAAALEHDKAYDRQLDSGDNPYLKYNHADAEFQERLKEDTSFGGNLGRA
VFQAKKRVLEPLGLVEEPVKTAPGKKRPVEHSPVEPDSSSGTGKAGQQPARKRLNFGQTG
DADSVPDPQPLGQPPAAPSGLGTNTMATGSGAPMADNNEGADGVGNSSGNWHCDSTW
MGDRVITTSTRTWALPTYNNHLYKQISSQSGASNDNHYFGYSTPWGYFDFNRFHCHFSPR
DWQRLINNNWGFRPKRLNFKLFNIQVKEVTQNDGTTTIANNLTSTVQVFTDSEYQLPYVL
GSAHQGCLPPFPADVFMVPQYGYLTLNNGSQAVGRSSFYCLEYFPSQMLRTGNNFTFSYT
FEDVPFHSSYAHSQSLDRLMNPLIDQYLYYLSRTNTPSGTTTQSRLQFSQAGASDIRDQSR
NWLPGPCYRQQRVSKTSADNNNSEYSWTGATKYHLNGRDSLVNPGPAMASHKDDEEKF
FPQSGVLIFGKQGSEKTNVDIEKVMITDEEEIRTTNPVATEQYGSVSTNLQRGNRQAATAD
VNTQGVLPGMVWQDRDVYLQGPIWAKIPHTDGHFHPSPLMGGFGLKHPPPQILIKNTPVP
ANPSTTFSAAKFASFITQYSTGQVSVEIEWELQKENSKRWNPEIQYTSNYNKSVNVDFTVD
TNGVYSEPRPIGTRYLTRNL

**Figure S1. The respective positions of the peptides are highlighted.**

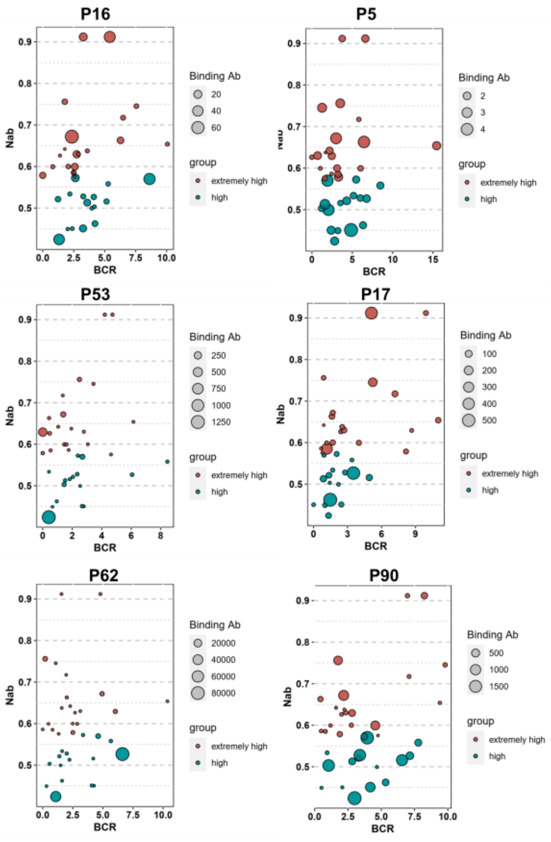


**Figure S2.** The epitopes of peptides, were assessed for Nab, Bab, and BCR recognition in both serum and PBMC samples obtained from hemophilia A/B patients with high or extremely high Nab titers. The calculated epitope profiles were presented.

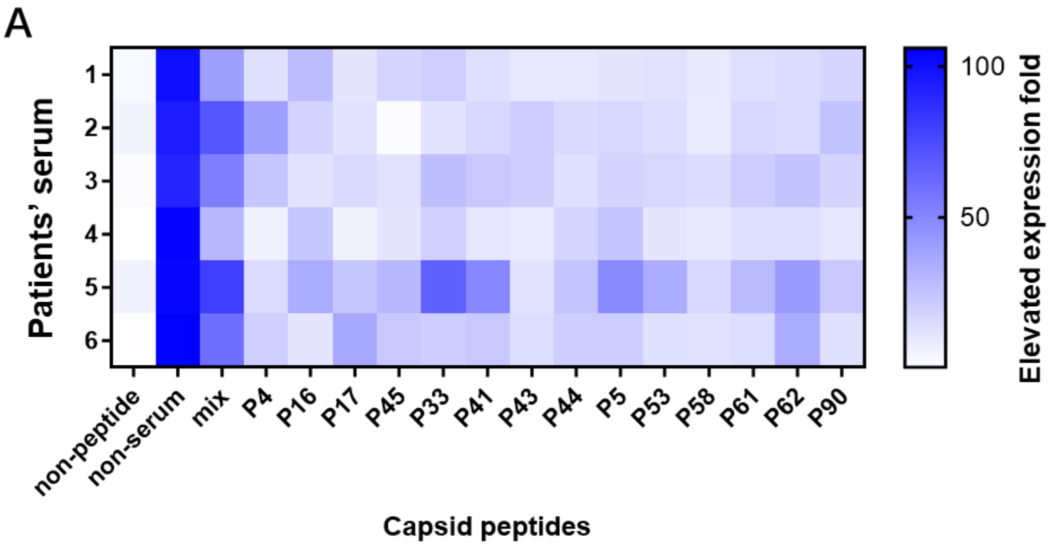


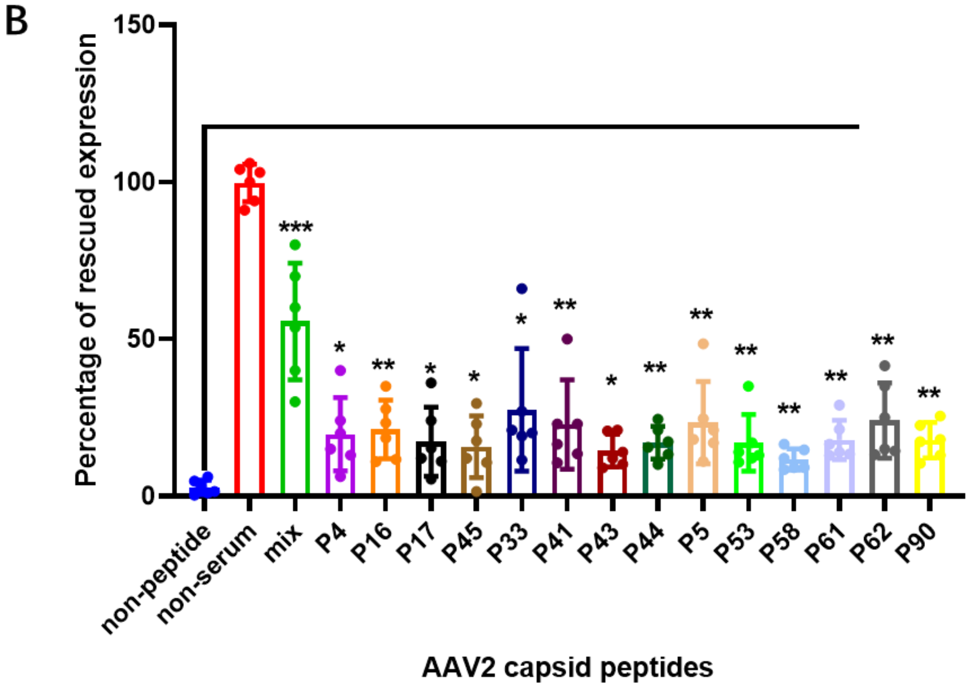


**Figure S3. The inhibitory potential of epitope peptides was assessed using an in vitro experiment of AAV gene transduction to HEK293 cells.** (**A&B**) In this model, compared with non-peptides-incubated group,the fold change of the rescued expression in luciferase activity was measured and analyzed to determine the extent of blocking achieved when serum (1 μL per well in 96-well plate) with the extremely high Nab titer to AAV2 (dilution > 1:128) was pretreated with single peptide (1 μg/mL) or a mixture

of epitope peptides (1 μg/mL), and then 1e8 vg AAV2-luc particles were added in the mixture, finally the serum-peptide-AAV2 mixture were added in the supernatant of HHEK293 cells.

| Dilution | serum-1 | serum-2 | serum-3 | serum-4 | serum-5 | serum-6 | serum-7 | serum-8 | serum-9 |
|---|---|---|---|---|---|---|---|---|---|
| 1:1 | 354 | 669 | 765 | 351 | 250 | 558 | 226 | 355 | 655 |
| 1:2 | 563 | 859 | 946 | 691 | 774 | 767 | 441 | 618 | 783 |
| 1:4 | 1353 | 2816 | 2139 | 1945 | 720 | 2034 | 1004 | 2832 | 1525 |
| 1:8 | 4209 | 3643 | 5395 | 6390 | 4611 | 5331 | 2050 | 4540 | 8651 |
| 1:16 | 7820 | 8554 | 8257 | 7209 | 8846 | 13949 | 9231 | 8551 | 9767 |
| 1:32 | 17545 | 15984 | 19763 | 10260 | 9481 | 22427 | 18364 | 13409 | 13905 |
| 1:64 | 28731 | 28779 | 25332 | 26252 | 24221 | 29061 | 25721 | 19006 | 23643 |
| 1:128 | 46146 | 39128 | 32871 | 30789 | 27076 | 33025 | 32976 | 32079 | 28898 |
| 1:256 | 42445 | 42697 | 44300 | 43886 | 32868 | 40135 | 46999 | 40570 | 38370 |
| 1:512 | 54453 | 51438 | 44695 | 44629 | 40031 | 47774 | 50022 | 50321 | 47301 |
| no-serum | 52204 | 54662 | 51074 | 58745 | 50577 | 51637 | 53846 | 57007 | 56365 |

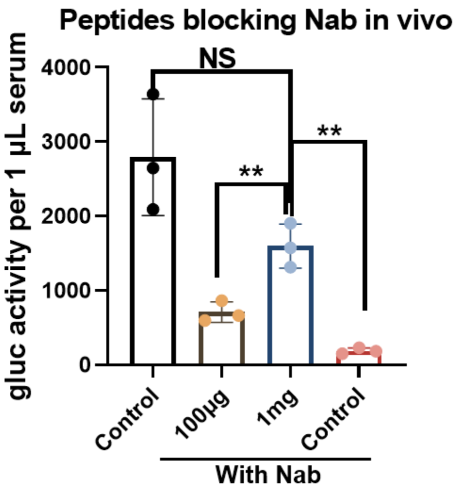


**Figure S4.** The female C57 mice, aged 6-8 weeks, were selected and retro-orbitally injected AAV2 virus particles (AAV2-GFP, 1e10 vg per mouse) into the mice. After one week, we collected serum from the mice and determined the titer of AAV2 neutralizing antibodies through in vitro neutralizing antibody assay. The neutralizing antibody titer against AAV2 in peripheral blood serum of the mice reached 1:64 (using the same Nab assay method as in the manuscript). Subsequently, we injected the entire peptide mixture (100μg or 1mg per mouse in 300 μl PBS) intravenously into the mice. One hour later, we intravenously injected 1e11 vg AAV2-Gluc (Gaussia Luciferase) per mouse, and on the 7th day, we collected venous blood from the mice to assess luciferase activity.

**Table S1.** The peptides were from the AAV2 capsid amino acid sequence that can recognized by the AAV2 neutralizing antibody, A20.

| seq | length | id |
|---|---|---|
| DWLEDTLSEGIRQWWKLKPG | 20 | P4 |
| EGIRQWWKLKPGPPPPKPAE | 20 | P5 |
| KEDTSFGGNLGRAVFQAKKR | 20 | P16 |
| NLGRAVFQAKKRVLEPLGLV | 20 | P17 |
| ALPTYNNHLYKQISSQSGAS | 20 | P33 |
| GFRPKRLNFKLFNIQVKEVT | 20 | P41 |
| KEVTQNDGTTTIANNLTSTV | 20 | P43 |
| TTTIANNLTSTVQVFTDSEY | 20 | P44 |
| TSTVQVFTDSEYQLPYVLGS | 20 | P45 |
| QMLRTGNNFTFSYTFEDVPF | 20 | P53 |
| PLIDQYLYYLSRTNTPSGTT | 20 | P58 |
| AGASDIRDQSRNWLPGPCYR | 20 | P61 |
| CYRQQRVSKTSADNNNSEYS | 20 | P62 |
| EIQYTSNYNKSVNVDFTVDT | 20 | P90 |
