# Supplementary material for: An investigation of the immune epitopes of adeno-associated virus capsid-derived peptides among hemophilia patients
Source: Mol Ther Methods Clin Dev. 2024 Apr 2;32(2):101245. doi: 10.1016/j.omtm.2024.101245 (PMC11039395; doi:10.1016/j.omtm.2024.101245)
Supplement: Document S2. Article plus supplemental information [file mmc2.pdf]

Molecular Therapy
Methods & Clinical Development
Original Article

An investigation of the immune epitopes of adeno-associated virus capsid-derived peptides among hemophilia patients

Li Liu,[1,2,4] Bingqi Xu,[1,2,4] Lingling Chen,[1,2] Jia Liu,[1,2] Wei Liu,[1,2] Feng Xue,[1,2] Sizhou Feng,[1,2] Erlie Jiang,[1,2] Mingzhe Han,[1,2] Wenwei Shao,[3] Lei Zhang,[1,2] and Xiaolei Pei[1,2]

[1]State Key Laboratory of Experimental Hematology, National Clinical Research Center for Blood Diseases, Haihe Laboratory of Cell Ecosystem, Institute of Hematology & Blood Diseases Hospital, Chinese Academy of Medical Sciences & Peking Union Medical College, Tianjin 300020, P.R. China; [2]Tianjin Institutes of Health Science, Tianjin 300020, P.R. China; [3]Academy of Medical Engineering and Translational Medicine, Tianjin University, Tianjin 300072, P.R. China

Adeno-associated virus (AAV) is an optimal gene vector for monogenic disorders. However, neutralizing antibodies (Nabs) against AAV hinder its widespread application in gene therapy. In this study, we biosynthesized peptides recognized by the binding antibodies (Babs) from the sera containing high Nab titers against AAV2. We established four immunological methods to detect immune epitopes of the AAV2-derived peptides, including a Bab assay, Nab assay, B cell receptor (BCR) detecting assay, and immunoglobin-producing B cell enzyme-linked immunosorbent spot (B cell ELISpot) assay. Correlations among the epitopes determined by these four methods were analyzed using the serum samples and peripheral blood mononuclear cells (PBMC) from 89 patients with hemophilia A/B. As decoys, the peptides' ability to block the Nab of AAV2 particles was assessed using AAV transduction models both *in vitro* and *in vivo*. Overall, we provide insights into AAV2-capsid-derived peptide immune epitopes, involving the Nab, Bab, BCR, and B cell ELISpot assays, offering alternative immunological evaluation approaches and strategies to overcome Nab barriers in AAV-mediated gene therapy.

INTRODUCTION

The existence of antibodies against adeno-associated virus (AAV) in human serum presents a significant hurdle to the clinical application of AAV. These antibodies, known as neutralizing antibodies (Nabs), attach to the AAV capsid, impeding the entry of AAV particles into target cells. Detecting Nabs through classical cell culture methods or rapid cell-binding methods is crucial before AAV-mediated gene therapy in clinical trials.[1,2] Patients with a low level of AAV Nabs (dilution ratio of the half-maximal inhibitory concentration between 1:1 and 1:8) in serum have exhibited successful gene transduction,[3] a phenomenon also linked to positive binding antibody (Bab) responses against AAV.[4] Given the variability in B cell receptor (BCR) and immunoglobulin (Ig) repertoires among individuals, along with the potential impact of antibody-mediated immune responses on AAV gene therapy efficacy, there is a pressing need for additional immune evaluation methods. These methods aim to assess the immune status of patients before and after AAV gene therapy, providing a comprehensive understanding of their gene-therapy-specific immune responses.

Primates, including humans, serve as natural hosts for AAV, resulting in a prevalence of Nabs or Babs against AAV among individuals. AAV2, a primary serotype of wild-type AAVs, is notably reported to have the highest prevalence of Nabs among all AAV serotypes.[5–7] In contrast to T cell-mediated immune responses, the immune response involving Ig hinders AAV particles extracellularly, rather than intracellularly, during administration. The epitopes on the AAV capsid that interact with specific antibodies are distributed both on the surface and inside AAV particles.[8,9] Previous research indicates that the binding sites of Nabs on AAV overlap with the binding site of AAVR and empty AAV particles can block Nab and enhance transduction.[9,10] Despite successful methods for predicting BCR epitopes in various diseases, methods specifically for predicting BCR epitopes related to AAVs are still lacking. The relationship between BCR epitopes, Nab/Bab epitopes, and the activity of Ig-producing memory B cells of AAVs is not fully understood.

Received 25 May 2023; accepted 29 March 2024;
https://doi.org/10.1016/j.omtm.2024.101245.

[4]These authors contributed equally

Correspondence: Wenwei Shao, Academy of Medical Engineering and Translational Medicine, Tianjin University, Tianjin 300072, P.R. China.
E-mail: wenwei.shao@tju.edu.cn
Correspondence: Lei Zhang, State Key Laboratory of Experimental Hematology, National Clinical Research Center for Blood Diseases, Haihe Laboratory of Cell Ecosystem, Institute of Hematology & Blood Diseases Hospital, Chinese Academy of Medical Sciences & Peking Union Medical College, Tianjin 300020, P.R. China.
E-mail: zhanglei1@ihcams.ac.cn
Correspondence: Xiaolei Pei, State Key Laboratory of Experimental Hematology, National Clinical Research Center for Blood Diseases, Haihe Laboratory of Cell Ecosystem, Institute of Hematology & Blood Diseases Hospital, Chinese Academy of Medical Sciences & Peking Union Medical College, Tianjin 300020, P.R. China.
E-mail: peixiaolei@ihcams.ac.cn

This is an open access article under the CC BY license (http://creativecommons.org/licenses/by/4.0/).

Table 1. Characteristics of patients (*N* = 89)

Characteristic	Value
Age, year, mean (range)	29.47 (3–59)
Gender, *n* (%)	
Male	89 (100.00)
Diagnosis, *n* (%)	
HA	66 (74.16)
HB	23 (25.84)
FVIII/FIX activity, *n* (%)	
<1%	51 (72.86)
≥1%	19 (27.14)
Enrolled and infused with gene therapy, *n* (%)	
Yes	0 (0)
No	89 (100)
Hemophilia with inhibitors	
Yes	10 (11.24)
No	79 (88.76)
Duration of continuous infusion of FVIII/FIX, day, mean (range)	4,237.93 (182–11,680)

HA, hemophilia A; HB, hemophilia B; FVIII, factor VIII; FIX, factor IX.

In this study, we biosynthesized AAV-capsid-derived peptides previously reported to be recognized by Babs in human serum with a high titer of Nabs. We established four immune epitope detection methods including an enzyme-linked immunosorbent assay (ELISA) for detecting Babs, AAV transduction assay for detecting Nabs, flow cytometry assay for detecting BCRs on B cells, and B cell ELISpot for detecting AAV-recognizing, antibody-producing B cells. To assess BCR, Nab, Bab, and Ig production by activated B cells recognizing AAV or AAV-derived peptides, we collected serum and peripheral blood mononuclear cell (PBMC) samples from 89 patients diagnosed with hemophilia A/B. Four immunological assays were used to elucidate differences in identifying immunogenic epitopes of AAV capsid proteins and their correlation with Nabs. Finally, the blocking capability of the epitope peptides to Nabs during AAV2 transduction was evaluated through *in vitro* and *in vivo* experiments.

RESULTS

In the serum of patients with hemophilia without AAV gene therapy, there is a notably low correlation between the Bab recognizing AAV2-capsid-derived peptides and the Nab of AAV2

Hemophilia has been an early target for AAV gene therapy, with Nabs in patients with hemophilia posing primary obstacles to AAV gene transduction. To address this, peripheral blood sera and PBMCs were collected from 89 patients with hemophilia (Table 1), and AAV Nab assays on the sera samples were conducted through an *in vitro* experiment (see materials and methods). The results indicated that 56% of the patients exhibited extremely high (dilution ratio is >1:512) or high levels (dilution ratio is between 1:128 and 1:512), 13% had moderate levels (dilution ratio is between 1:32 and 1:128), and 34% had low (dilution ratio is between 1:8 and 1:32) or very low levels (dilution ratio is between 1:1 and 1:8; Figure 1A) of Nabs.

Based on previous reports, the amino acid sequence of the AAV2 capsid protein contains multiple peptide epitope sequences recognized by antibodies in the serum.[11] Following this sequence, we synthesized 14 peptides, each with a length of 20 amino acids (Table S1). The peptides were marked on the crystal structure of the AAV2 viral particle (in colors, Figure 1B), but this does not include P4, P5, P16, or P17, for they were located on the VP1/2 region[12] and the crystal structure of the AAV2 particle was based on VP3. The 14 peptides were initially evaluated in the sera of the 89 patients with hemophilia using the direct ELISA (see materials and methods). The results indicated significantly higher Bab levels recognizing the P16, P17, P33, P41, P44, P45, P58, P62, and P90 peptides, while P4, P5, P53, and P61 did not show such elevation (Figure 1C).

Despite these peptides all deriving from the amino acid sequence of the AAV2 capsid protein, the consistency of their immunogenicity in patients with hemophilia remains to be explored. Calculating the correlation among the peptide epitopes based on the peptide-recognized Bab levels varied in the sera samples, Pearson's score revealed generally low associations among most of the peptides but a notably high correlation between P33 and P58 peptide epitopes (Figure 1D). The Bab levels recognizing the peptides in the sera of the 89 patients were presented using a heatmap, alongside the subgroups classified by the Nab levels (Figure 1E). Interestingly, samples with higher levels of Babs recognizing the P44 peptide tended to have lower levels of antibodies recognizing the P58 peptide, and vice versa. This suggests significant heterogeneity in Babs recognizing the different peptides in patient sera.

Additionally, we calculated the correlation between the Nab blocking AAV2 transduction and the Bab recognizing the single peptide in the sera of the 89 patients using the Pearson's score. The results showed a low correlation among them (Figure 1F). The above findings suggest that Bab detection by these peptides in serum does not effectively reflect Nab levels.

In PBMCs from patients with hemophilia with high Nab levels, the BCR recognizing the peptide epitopes shows a strong correlation with Nab levels

Initially, as depicted in Figure 2A, we established a BCR labeling method to fluorescently tag the BCRs on B cells capable of recognizing epitope peptides or AAV viral particles, along with a gating strategy. PBMCs were selected from patients with extremely high or high Nab levels, and flow cytometry was performed using the BCR labeling method. Compared to the control groups, the proportions of B cells recognizing individual peptides or AAV2/AAV8 full particles were significantly higher. The top four highest-prevalence peptides recognized by the BCRs on B cells in the individual PBMC samples were P44, P53, P62, and P41 (Figure 2B). Additionally, the proportion

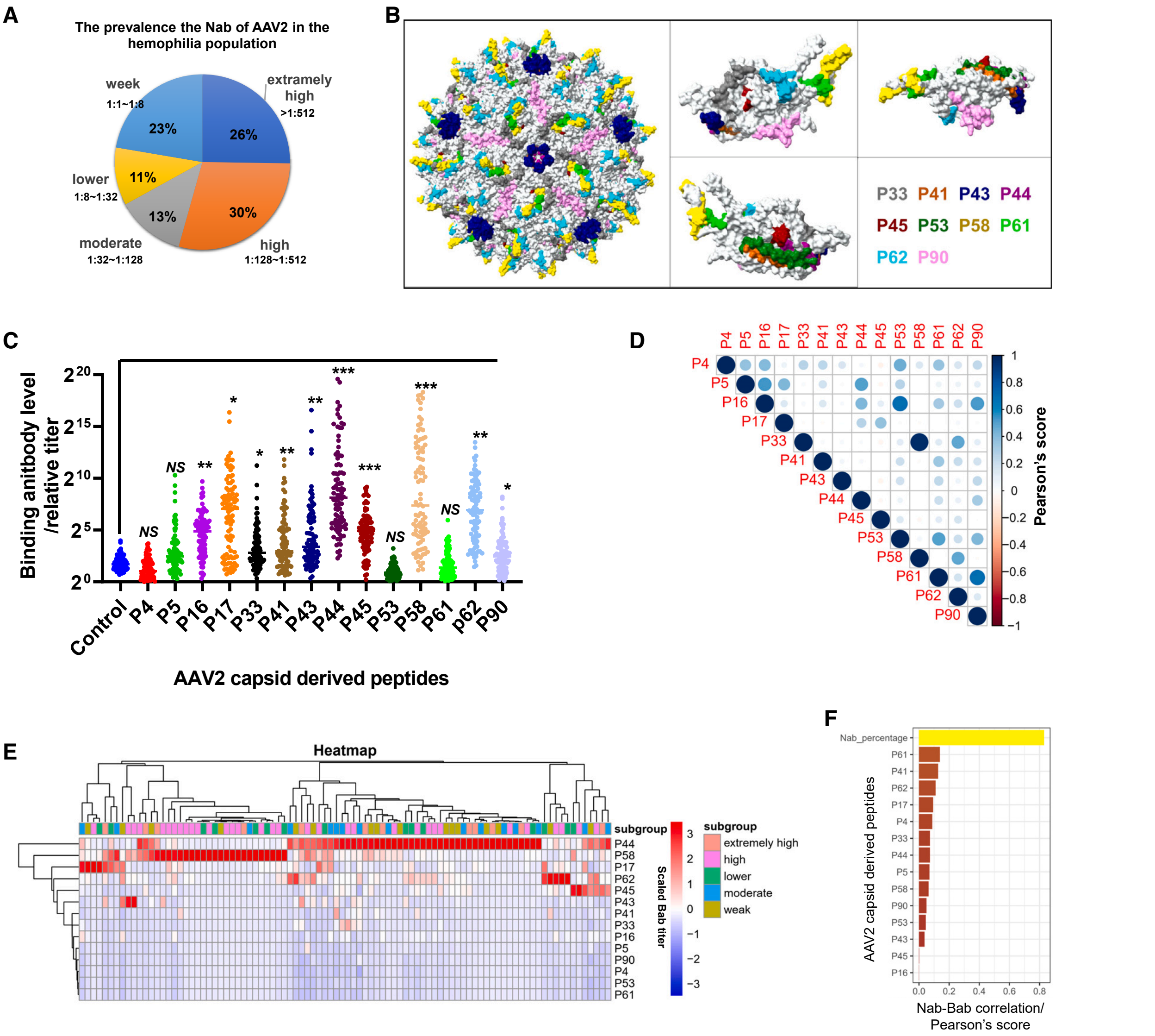

Figure 1. The analysis of Babs in the serum of patients with hemophilia A/B targeting the peptides was conducted

(A) The prevalence of Nabs to AAV2 was summarized in 89 serum samples obtained from patients with hemophilia determined by the Nab assay. (B) The location of the epitope peptides on the assembled AAV2 particle (PDB: 6IH9) and single capsid are highlighted. (C) Directed ELISA was employed to measure the Bab titer in the serum samples against the epitope peptides, and each rectangle represents the serum from one individual patient. (D) The correlation among the epitope peptides recognized by the Babs in the serum samples was calculated by Pearson's score. (E) A heatmap was utilized to display the scaled titer (the OD_{450} readings are scaled to range from −3 to 3) of the Babs to the peptides in the serum samples, along with the corresponding Nab subgroups. (F) The correlation among the individual peptides (calculated by scaled titer) and the Nabs (calculated by the dilution ratio) was analyzed using Pearson's score according to their level of variation in the serum samples.

of B cells directly recognizing AAV2 and AAV8 viral particles did not significantly increase compared to the single peptides (Figure 2B).

By comparing the correlation (Pearson's score) between individual peptides recognized by BCRs on B cells from patients with hemophilia, we found a high correlation among the peptides (Figure 2C). The proportions of BCR-expressing B cells recognizing peptides or AAV viral particles in the PBMCs of 36 patients with hemophilia with high or extremely high Nabs were also depicted using a heatmap showing considerable heterogeneity in B cells recognizing different peptides among the patients (Figure 2D). Furthermore, the correlations between the proportions of BCR-expressing B cells recognizing individual peptide or AAV particles in the peripheral blood B cells of 36 patients and Nabs were calculated by Pearson's score. The peptide P61, which is located in the "spike" region of the AAV2 virus, was found to have the highest correlation with Nabs (Figure 1F). These results indicate a strong correlation between BCR-expressing B cells recognizing the peptides and the Nabs blocking AAV2 transduction. As the main source of

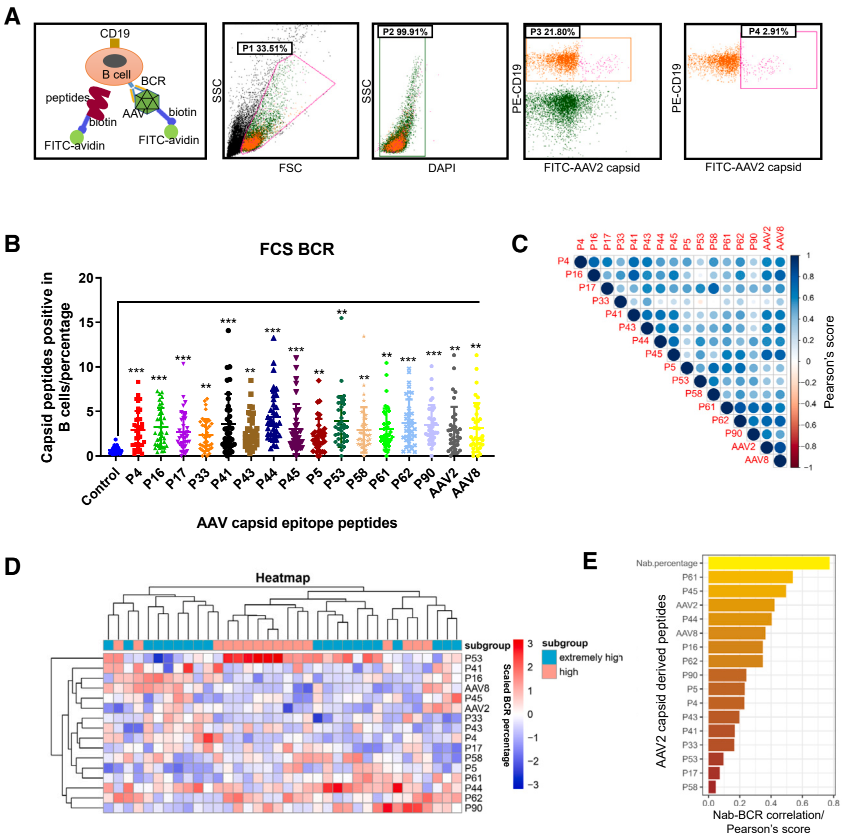

Figure 2. The BCR expressed on B cells was labeled with epitope peptides derived from the AAV2 capsid, and the prevalence was assessed using the antigen labeling system

(A) The construction of the BCR-antigen labeling system and the gating strategy employed in this assay are presented. (B) B cells in the PBMCs from patients with hemophilia (from 1 mL peripheral blood and suspended with 100 μL PBS) were labeled with different peptides (1 μg/mL) or AAV particles (1e8 vg/mL) and quantified using flow cytometry. (C) Correlation analysis (Pearson's score) was performed to examine the relationship between the BCR epitopes (the percentage of positively antigen-recognizing B cells in total $CD19^+$ B cells) of different peptides and AAV particles in patients with hemophilia. (D and E) Heatmap analysis was conducted to investigate the percentage of BCR-positive cells recognizing the peptides and the Nab titer among individual patients with hemophilia, and each rectangle represents an individual patient (D), along with their correlation (Pearson's score) with the Nab titer (calculated by the dilution ratio) in the corresponding serum (E).

Nab-produced plasma cells, peripheral B cells express a BCR that has high correlation in recognizing peptides and AAV particles, supporting the hypothesis that Nabs can recognize linear peptide epitopes.

The correlation among the four immunological components in the sera or PBMCs of patients with hemophilia—Nab, Bab, BCR, and memory B cells—was analyzed by detecting the peptides or AAV particles

Based on the epitope detection of the peptides by Babs, Nabs, and BCRs, a positive linear correlation was observed between BCRs and Nabs, particularly when detecting P61, P44, and P4, while the correlation between Nabs and Babs seemed low (Figures 3A and S2). Furthermore, a B cell ELISpot assay was established (Figure 3B) to measure AAV2-capsid-specific antibody-secreting cells in the PBMCs of patients with hemophilia.[13] The correlations among Nabs, AAV2-specific BCR, and AAV2-specific IgG-producing B cells from peripheral blood samples of the patients were assessed using Pearson's correlation coefficient. As depicted in Figures 3C and 3D, the B cell ELISpot displayed a lower correlation with Nabs and BCRs when immunologically detecting AAV2 particles. Lastly, the correlations between the four detection methods (Nabs, Babs, BCRs, and B cell ELISpot) in detecting the peptides or AAV particles were calculated by the Pearson's score and represented by the "distance" (distance = 1 – the Pearson's score), and BCR detection exhibited the closest proximity to Nab detection in patients with hemophilia (Figure 3E).

The capacity of the peptides to block the Nab in the serum of patients with hemophilia and AAV2-immunized mice was evaluated

The presence of Nabs in serum poses a significant obstacle during AAV transduction, while the Babs, BCRs, and antibody-secreting B cells might be involved in the immune response post-AAV transduction. In this study, we evaluated the capacity of the epitope peptides blocking Nabs both *in vitro* and *in vivo*. Initially, serum samples containing high titers of Nabs to AAV2 were preincubated with individual peptides or mixed peptides and then incubated with AAV2-luciferase (Luc) particles before being added to the supernatant of Huh7 cells or HEK293 cells (Figure 4A). As summarized in Figures 4B, 4C, and S3, both the single peptides and the mixture exhibited different capabilities in blocking Nabs from different patients with hemophilia in Huh7 cells and HEK293 cells. It is noteworthy that the efficiency of peptide blocking varied among patients with hemophilia A/B, and the ratio of the peptides blocking Nab should be rationally optimized. Additionally, we used peptide mixtures at different doses to block Nabs in the serum or the circulating of AAV2-immunized C57 mice and observed a dose-dependent blocking effect of the peptide mixture both *in vitro* and *in vivo* (Figures 4D–4F). This finding provides a promising strategy for AAV gene therapy in patients with high levels of Nabs to AAV.

DISCUSSION

In our study, drawing from previous reports, we redesigned and synthesized peptides derived from AAV2 capsid. Peripheral blood serum was collected from 89 patients with hemophilia, and we established four antibody detection methods targeting AAV2. The differences and similarities of these four methods in immune responses when

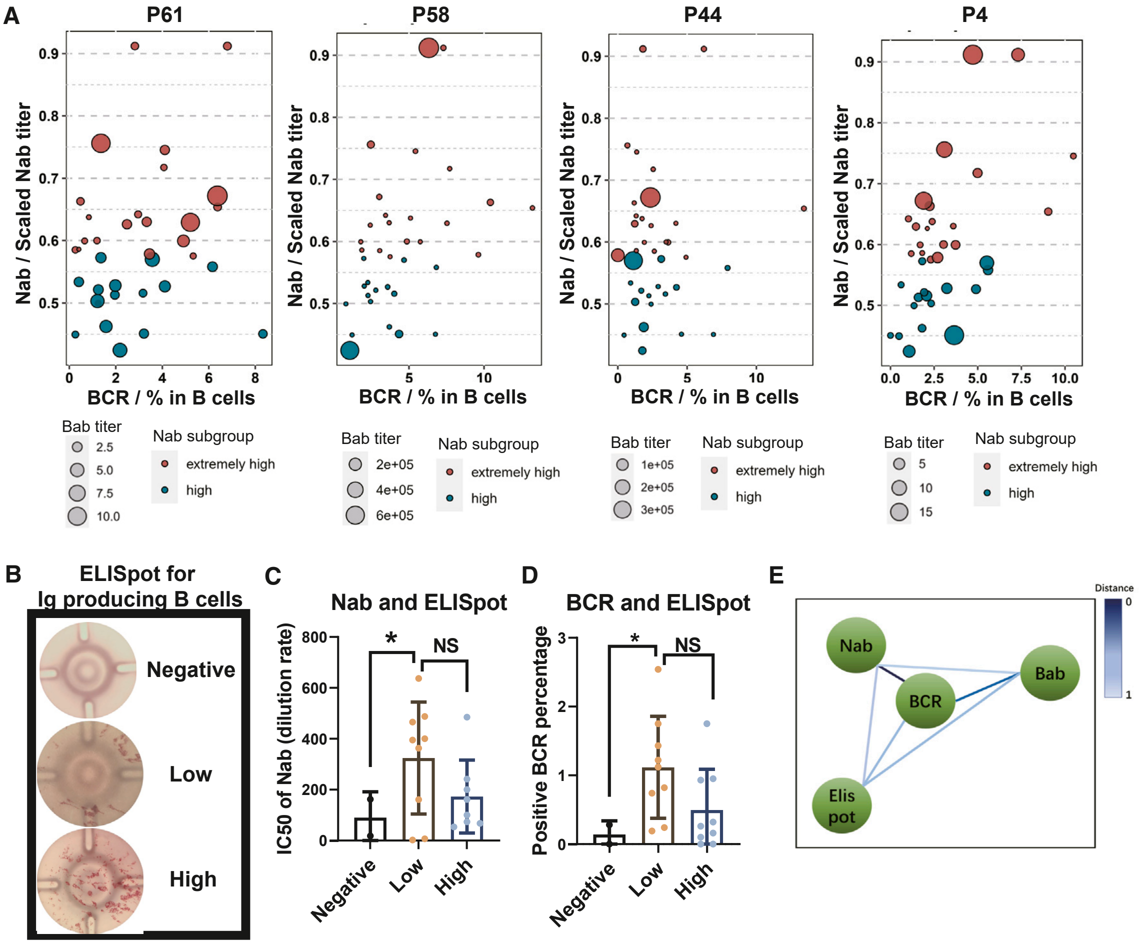

Figure 3. A comparison was conducted to determine the distance between Nabs, Babs, BCRs, and B cell ELISpot in the detection of AAV and its derived peptides

(A) The correlation among Nabs (calculated by dilution ratio), Babs (calculated by scaled titer), and BCRs (calculated by cell percentage) from the 36 patients with hemophilia in the extremely high or high Nab groups, to recognize four representative epitope peptides, was calculated using Pearson's correlation coefficient and is presented. (B) The B cells producing the Ig to recognize the epitope peptides or AAV particles were stained using the ELISpot assay to detect the Ig plots, and the results of B cell ELISpot were categorized into three groups: negative, low spot count, and high spot count, as shown representatively. (C and D) In the serum sample from the three B cell ELISpot groups, the corresponding Nabs (calculated by dilution ratio) against AAV2 in the serum and the percentage of BCRs recognizing AAV2 were statistically analyzed. (E) The distance between Nabs, Babs, BCRs, and Ig-producing B cells was calculated (distance = 1 − r) based on the recognizing ability of AAV particles or epitope peptides among the patients with hemophilia.

recognizing AAV2 or AAV2-derived peptides were compared. Finally, through *in vitro* experiments, we validated the peptides' ability to block Nabs against AAV2.

It is widely acknowledged that Nab possess the capability to identify and engage with the three-dimensional epitopes of a specific protein, thereby impeding its functionality. However, it should be noted that the scope of recognition by Nab extends beyond the protein itself, as they can also identify peptides that harbor identical amino acid sequences to the binding site recognized by Nab on the protein. This implies that Nab may exhibit cross-reactivity towards peptides sharing sequence homology with the epitopes targeted on the protein surface. As mentioned earlier, peptides containing the corresponding linear epitopes may be recognized by these Nabs.[14,15] It is currently acknowledged that the P33 and P90 peptides are located at the binding sites of Nabs, such as A20, and the "plateau"-like region of the AAV2 virus.[16] Asokan's team achieved the evasion of Nabs during the gene therapy process by employing an enzyme to degrade them and developed novel AAV vectors capable of effectively escaping Nabs through the analysis of antigenic epitopes on the AAV surface and the application of an evolutionary model.[17–19]

According to the study by Maheshri et al., amino acid residues capable of effectively evading Nabs were identified through the use of AAV mutant libraries and a Nab evolution model.[20] Up to 12 residues were identified, distributed across the VP1, VP2, and VP3

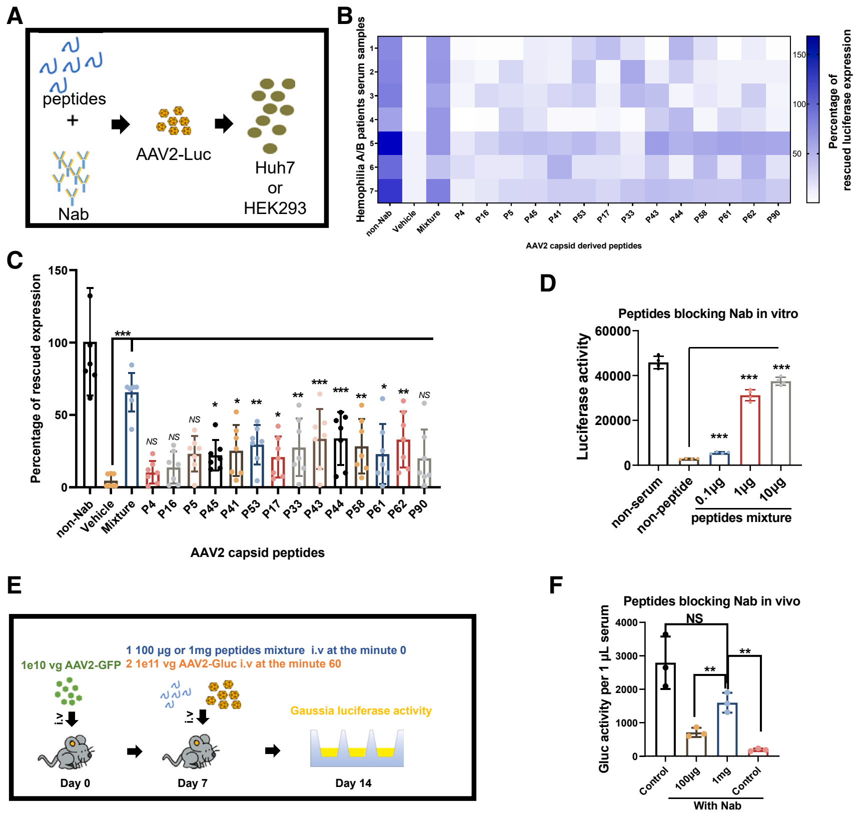

Figure 4. The inhibitory potential of epitope peptides to Nab was assessed both *in vitro* and *in vivo*

(A) In the *in vitro* experiment, the serum (1 μL per well in 96-well plate) with the high Nab titer against AAV2 (dilution ratio > 1:128) that was pretreated with a single peptide (1 μg/mL) or a mixture of epitope peptides (1 μg/mL) for 30 min. Subsequently, 1e8 vg AAV2-luciferase (Luc) particles were added to the mixture for another 30 min, and finally, the serum-peptide-AAV2 mixture were added in the supernatant of Huh7 cells or HEK293 cells and the Luc activity was measured after 48 h. (B and C) In the *in vitro* experiment, the percentage of the rescued expression in Luc activity in Huh7 cells by the peptides blocking Nabs was measured and analyzed. (D) The peptide mixture at concentrations of 0.1, 1, and 10 μg was preincubated with the Nab-containing serum (1 μL) for 30 min, and subsequently, the blocking efficacy of the peptide mixture on Nabs at different doses was assessed on Huh7 cells. (E) In the *in vivo* experiment, a dose of 1e10 vg AAV2-GFP particles was intravenously injected into the C57 mice. After 1 week, serum from the mice was collected, and the Nab was determined. Subsequently, the peptide mixture (100 μg or 1 mg per mouse in 300 μL PBS) was intravenously injected into the mice. One hour later, a dose of 1e11 vg AAV2-Gluc (Gaussia Luc) particles was intravenously injected into the mice, and 7 days later, venous blood from the mice was collected to assess luciferase activity. (F) The Nab-blocking capability of the peptide mixture at the dose of 100 μg or 1 mg per mouse was measured and statistically analyzed.

regions,[20] and the peptides in this study covered 8 of these sites. Huttner et al.'s research on loop sites in AAV2 capsid proteins revealed 6 peptide-insertable loop sites.[21] Inserting peptides into 2 of these sites significantly enhanced gene delivery efficiency,[21] and these two sites were not covered in our study. In Maersch et al.'s study, joint mutations at positions 449, 458, 459, 493, and 551 in AAV2 capsid proteins led to clones with significant alterations in tropism and transduction efficiency.[22] Some mutations resulted in the loss of antibody binding ability, highlighting the importance of these sites as Nab binding sites,[22] and the peptides in our study covered three of the six positions. Considering these findings, we recognize the diverse nature of Nab binding sites. When using virus-derived peptides for Nab inhibition, precise adjustment of the peptide proportions is crucial for optimal blocking effect. Additionally, variations in Nab binding sites within different patients may exist in terms of binding locations and the quantity of antibodies corresponding to each site.

The correlation or distance among BCR, Ig-producing B cell, Bab, and Nab epitopes to the same peptides is complex, influenced not only by different amino acid sequences involved in recognition but also by their roles in immunity. The BCR/Ig linear epitope plays a crucial role in antigen presentation and activation of Ig-producing B cells.[23] In many cases, antibodies can recognize both linear epitopes and three-dimensional epitopes (referred to as "conformational epitopes") that share the same amino acid sequence.[24,25] It has been demonstrated that a length of twenty amino acids can represent the BCR/Ig epitope in most cases.[26,27]

In AAV gene therapy, Mingozzi's team first proposed using a decoy strategy to address Nabs, and they also engineered the capsid amino acid sequence to prevent their entry into the target cells, thereby reducing immunotoxicity.[10] The use of AAV empty capsids can effectively block over half of the Nabs. Since empty capsids share the same spatial epitopes as AAV full particles, they are undoubtedly the most efficient in terms of blocking effectiveness. However, empty capsids also pose some unknown issues. For instance, they can infect other cells in the host, increasing the burden on these cells and potentially triggering an immune response through the resulting antigen presentation process.[28,29]

Various approaches have been proposed to enhance AAV gene transduction efficiency.[30–33] Limited research has focused on overcoming secondary gene therapy by peptides' inhibition of Nabs. Short peptides, especially those shorter than 20 amino acids without a cross-linked carrier protein or adjuvant, are unlikely to induce an immune response and degrade rapidly.[34] If safety validation experiments confirm no significant toxicity issues with peptides, then they will become one of the viable choices for blocking Nabs. The current challenge primarily revolves around selecting peptide sequences and determining which sites to cover effectively, addressing all the

AAV-binding sites for Nabs. Optimal mixing ratios between peptides are also a key aspect under exploration.

MATERIALS AND METHODS

The collection of clinical samples and information

A collection of 89 serum samples and PBMC samples from patients diagnosed with hemophilia A/B was conducted. The execution of this study adhered to rigorous monitoring and scrutiny by the Ethics Committee of the Blood Diseases Hospital, Chinese Academy of Medical Sciences, in accordance with the principles outlined in the Declaration of Helsinki (approval no. CAMSCRF2021013-EC-2). Additionally, we confirm that informed consent was diligently obtained from all human research participants.

The detection of Bab against AAV

In this study, the direct ELISA method was utilized for the Bab assay. 1 μg peptide (biosynthesized and purified by NJPeptide, purity > 97%) was combined with 100 μL coating buffer and then applied to a 96-well ELISA plate overnight at 4°C. The plate underwent four washes with 300 μL PBS and was subsequently treated with 200 μL 5% BSA in PBS for 1 h at 37°C. Following five additional washes with 300 μL PBS, the plate was exposed to diluted serum (1:100 in this study) for 2 h at 37°C. After another round of washing (five times with 300 μL PBS), the plate was incubated with diluted horseradish peroxidase (HRP)-anti-human IgG antibody (1:2,000 in this study, Invitrogen #31410) for 1 h at 37°C. Finally, after a final wash (seven times with 300 μL PBS), the HRP activity was determined using a 3,3′5,5′-tetramethylbenzidine (Thermo Scientific #34028) kit.

The detection of Nab against AAV

The detailed protocol of this assay has been previously described.[5,35] Serum from patients or immunized mice (C57BL6/N, female, aged 6–8 weeks, from Huafukang Beijing) was separated from venous blood by centrifugation at 5,000 × *g* for 5 min, and 2 μL serum was added to 50 μL Dulbecco's modified Eagle medium (DMEM; Gibco #11965092)+10% fetal bovine serum (FBS; HyClone #SH3007103) in the wells of a 96-well plate (Thermo Scientific #165306) to create a 1:1 dilution sample. For serum dilution, 25 μL of the serum-medium mixture was taken from the 1:1 dilution sample and then combined with 25 μL DMEM+10% FBS to create a 1:2 dilution sample, with subsequent dilution samples prepared at ratios of 1:4 to 1:512. A dose of 50 μL DMEM+10% FBS containing 1e8 vg AAV2-Luc particles was added to each well, followed by incubation of the AAV particles and serum for 30 min at 37°C. Huh7 cells or HEK293 cells (5e6 cells suspended in 100 μL DMEM+10% FBS) were then added to each well and incubated for 48 h at 37°C. Finally, Luc expression by the cells was measured using a Luc activity kit.

The detection of AAV-specific BCRs on B cells

The AAV particles or peptides were biotinylated using biotin (Thermo Scientific #20217). Subsequently, PBMCs (1e5 cells) obtained from patients with hemophilia were incubated with the biotin-conjugated particles (1e8 vg) or peptides (10 ng) on ice for a duration of 30 min. After washing twice with PBS, the cells were incubated with PE-anti CD19 antibody (BioLegend #302207, 2 μL) and fluorescein isothiocyanate-avidin (BioLegend #405101, 1 μL) on ice for 15 min. Finally, B cells from patients with hemophilia, expressing BCRs capable of recognizing the AAV particles or peptides, were analyzed using cytometry.

The Ig-producing B cell ELISpot

The B cells were isolated from PBMC samples using magnetic beads conjugated with anti-CD19 antibody (Meltenyi #130-050-301). Subsequently, the isolated B cells were cultured *in vitro* for a period of 4 days using the B cell expansion medium (STEMCELL #100-0645_C). On day 3 of the B cell culture, AAV particles (1e9 vg per well) or AAV-sourced peptides (1 μg per well), diluted in PBS, were precoated on the bottom of a PVDF membrane 96-well plate (Millipore #MSIPS4510). Prior to precoating, the PVDF membrane was treated with 35% ethanol (15 μL per well) for 1 min and washed with deionized water four times. The precoated plate was then incubated at 4°C overnight. Following the precoating step, the B cells were added to the antigen precoated 96-well plate and cultured continuously with the B cell expansion medium for a duration of 72 h. After the culture period, the cells and supernatant were removed, and the PVDF membrane in the wells was washed twice with PBS. Subsequently, a blocking buffer (4% BSA+PBST) was added and incubated for 30 min at 37°C. Following the incubation, the PVDF membrane was washed four times with PBST and then incubated with HRP-conjugated rabbit anti-human IgG Fc fragment antibody (diluted at 1:5,000 with PBST) for 1 h at 37°C. The PVDF membrane was then washed five times with PBST. To visualize the spots on the PVDF membrane, the substrate specific for ELISpot, with HRP conjugation, was added to the wells. The reaction was subsequently stopped by adding deionized water. The spots on the PVDF membrane were analyzed using an ELISpot scanner.

The *in vitro* assay of peptides to block Nab

The peptides (1 μg) were combined with serum containing Nab (1 μL) and incubated for 30 min at 37°C. Subsequently, the peptide and serum mixture were mixed with AAV2-Gaussia-Luc particles (1e8 vg) for an additional 30 min at 37°C. The resulting mixture was then added to the supernatant of Huh7 or HEK293 cells at a multiplicity of infection of 2,000. After 48 h of transduction, the Gaussia-Luc activity in the supernatant of Huh7 or HEK293 cells was measured using a Gaussia-Luc detection kit (Thermo Scientific #16181).

The *in vivo* assay of peptides to block Nab

Female C57 mice, aged 6–8 weeks, were intravenously injected with 1e10 vg AAV2-GFP particles. Seven days later, venous serum was collected, and the induced Nab in serum was determined. The immunized mice were equally grouped based on Nab titer, and either 100 μg or 1 mg peptides in 300 μL PBS were intravenously injected into the mice. After 1 h, 1e11 vg AAV2-GLuc particles were intravenously injected. Gaussia luciferase activity was measured by the Gaussia

Luciferase Glow Assay Kit (Pierce #16160) at 7 days post-AAV2-GLuc injection.

Statistical analysis

In this study, statistical analysis was conducted to assess the significance of differences in mean values using Student's t test. The Pearson's correlation coefficient was employed to calculate the correlation or distance between parameters in different groups. Each experiment was performed independently, with repetition conducted more than three times. A p value of less than 0.05 was considered statistically significant. The following notation was used to indicate the significance level: $*p < 0.05$, $**p < 0.01$, and $***p < 0.001$.

DATA AND CODE AVAILABILITY

The datasets generated and analyzed during the current study are available as supplemental information.

SUPPLEMENTAL INFORMATION

Supplemental information can be found online at https://doi.org/10.1016/j.omtm.2024.101245.

ACKNOWLEDGMENTS

This work was supported by the National Natural Science Foundation of China (82072046 and 82101853), the Haihe Laboratory of Cell Ecosystem Innovation Fund (22HHXBSS00022), the CAMS Innovation Fund for Medical Sciences (CIFMS) (2022-12M-2-003, 2021-I2M-1-003, and 2022-I2M-C&T-A-015), the National Key Research and Development Program of China (2019YFA0110802 and 2021YFA1101603), and the Tianjin Natural Science Foundation (23JCZXJC00040). The collection and treatment of clinical samples from patients and the animal model were approved by the ethical committee of the Institute of Hematology and Blood Diseases Hospital, Chinese Academy of Medical Sciences (CAMSCRF2021013-EC-2).

AUTHOR CONTRIBUTIONS

L.L., B.X., and X.P. performed experiments; L.L., X.P., and J.L. analyzed results and made the figures; L.C., F.X., W.L., S.F., M.H., and E.J. collected the clinical samples; L.Z., X.P., and W.S. designed the research and wrote the paper; and all authors read and approved the final manuscript.

DECLARATION OF INTERESTS

The authors declare no competing interests.

REFERENCES

1. Louis Jeune, V., Joergensen, J.A., Hajjar, R.J., and Weber, T. (2013). Pre-existing anti-adeno-associated virus antibodies as a challenge in AAV gene therapy. Hum. Gene Ther. Methods *24*, 59–67. https://doi.org/10.1089/hgtb.2012.243.

2. Guo, P., Zhang, J., Chrzanowski, M., Huang, J., Chew, H., Firrman, J.A., Sang, N., Diao, Y., and Xiao, W. (2019). Rapid AAV-Neutralizing Antibody Determination with a Cell-Binding Assay. Mol. Ther. Methods Clin. Dev. *13*, 40–46. https://doi.org/10.1016/j.omtm.2018.11.007.

3. Mingozzi, F., and High, K.A. (2013). Immune responses to AAV vectors: overcoming barriers to successful gene therapy. Blood *122*, 23–36. https://doi.org/10.1182/blood-2013-01-306647.

4. Schulz, M., Levy, D.I., Petropoulos, C.J., Bashirians, G., Winburn, I., Mahn, M., Somanathan, S., Cheng, S.H., and Byrne, B.J. (2023). Binding and neutralizing anti-AAV antibodies: Detection and implications for rAAV-mediated gene therapy. Mol. Ther. *31*, 616–630. https://doi.org/10.1016/j.ymthe.2023.01.010.

5. Pei, X., Shao, W., Xing, A., Askew, C., Chen, X., Cui, C., Abajas, Y.L., Gerber, D.A., Merricks, E.P., Nichols, T.C., et al. (2020). Development of AAV Variants with Human Hepatocyte Tropism and Neutralizing Antibody Escape Capacity. Mol. Ther. Methods Clin. Dev. *18*, 259–268. https://doi.org/10.1016/j.omtm.2020.06.003.

6. Boutin, S., Monteilhet, V., Veron, P., Leborgne, C., Benveniste, O., Montus, M.F., and Masurier, C. (2010). Prevalence of serum IgG and neutralizing factors against adeno-associated virus (AAV) types 1, 2, 5, 6, 8, and 9 in the healthy population: implications for gene therapy using AAV vectors. Hum. Gene Ther. *21*, 704–712. https://doi.org/10.1089/hum.2009.182.

7. Ling, C., Wang, Y., Feng, Y.L., Zhang, Y.N., Li, J., Hu, X.R., Wang, L.N., Zhong, M.F., Zhai, X.F., Zolotukhin, I., et al. (2015). Prevalence of neutralizing antibodies against liver-tropic adeno-associated virus serotype vectors in 100 healthy Chinese and its potential relation to body constitutions. J. Integr. Med. *13*, 341–346. https://doi.org/10.1016/S2095-4964(15)60200-X.

8. Tseng, Y.S., and Agbandje-McKenna, M. (2014). Mapping the AAV Capsid Host Antibody Response toward the Development of Second Generation Gene Delivery Vectors. Front. Immunol. *5*, 9. https://doi.org/10.3389/fimmu.2014.00009.

9. Meyer, N.L., and Chapman, M.S. (2022). Adeno-associated virus (AAV) cell entry: structural insights. Trends Microbiol. *30*, 432–451. https://doi.org/10.1016/j.tim.2021.09.005.

10. Mingozzi, F., Anguela, X.M., Pavani, G., Chen, Y., Davidson, R.J., Hui, D.J., Yazicioglu, M., Elkouby, L., Hinderer, C.J., Faella, A., et al. (2013). Overcoming pre-existing humoral immunity to AAV using capsid decoys. Sci. Transl. Med. *5*, 194ra92. https://doi.org/10.1126/scitranslmed.3005795.

11. Moskalenko, M., Chen, L., van Roey, M., Donahue, B.A., Snyder, R.O., McArthur, J.G., and Patel, S.D. (2000). Epitope mapping of human anti-adeno-associated virus type 2 neutralizing antibodies: implications for gene therapy and virus structure. J. Virol. *74*, 1761–1766. https://doi.org/10.1128/jvi.74.4.1761-1766.2000.

12. Wobus, C.E., Hügle-Dörr, B., Girod, A., Petersen, G., Hallek, M., and Kleinschmidt, J.A. (2000). Monoclonal antibodies against the adeno-associated virus type 2 (AAV-2) capsid: epitope mapping and identification of capsid domains involved in AAV-2-cell interaction and neutralization of AAV-2 infection. J. Virol. *74*, 9281–9293. https://doi.org/10.1128/jvi.74.19.9281-9293.2000.

13. Shah, H.B., and Koelsch, K.A. (2015). B-Cell ELISPOT: For the Identification of Antigen-Specific Antibody-Secreting Cells. Methods Mol. Biol. *1312*, 419–426. https://doi.org/10.1007/978-1-4939-2694-7_42.

14. Emini, E.A., Jameson, B.A., and Wimmer, E. (1983). Priming for and induction of anti-poliovirus neutralizing antibodies by synthetic peptides. Nature *304*, 699–703. https://doi.org/10.1038/304699a0.

15. Pantarotto, D., Partidos, C.D., Hoebeke, J., Brown, F., Kramer, E., Briand, J.P., Muller, S., Prato, M., and Bianco, A. (2003). Immunization with peptide-functionalized carbon nanotubes enhances virus-specific neutralizing antibody responses. Chem. Biol. *10*, 961–966. https://doi.org/10.1016/j.chembiol.2003.09.011.

16. McCraw, D.M., O'Donnell, J.K., Taylor, K.A., Stagg, S.M., and Chapman, M.S. (2012). Structure of adeno-associated virus-2 in complex with neutralizing monoclonal antibody A20. Virology *431*, 40–49. https://doi.org/10.1016/j.virol.2012.05.004.

17. Elmore, Z.C., Oh, D.K., Simon, K.E., Fanous, M.M., and Asokan, A. (2020). Rescuing AAV gene transfer from neutralizing antibodies with an IgG-degrading enzyme. JCI Insight *5*, e139881. https://doi.org/10.1172/jci.insight.139881.

18. Tse, L.V., Klinc, K.A., Madigan, V.J., Castellanos Rivera, R.M., Wells, L.F., Havlik, L.P., Smith, J.K., Agbandje-McKenna, M., and Asokan, A. (2017). Structure-guided evolution of antigenically distinct adeno-associated virus variants for immune evasion. Proc. Natl. Acad. Sci. USA *114*, E4812–E4821. https://doi.org/10.1073/pnas.1704766114.

19. Emmanuel, S.N., Smith, J.K., Hsi, J., Tseng, Y.S., Kaplan, M., Mietzsch, M., Chipman, P., Asokan, A., McKenna, R., and Agbandje-McKenna, M. (2022). Structurally

Mapping Antigenic Epitopes of Adeno-associated Virus 9: Development of Antibody Escape Variants. J. Virol. *96*, e0125121. https://doi.org/10.1128/JVI.01251-21.

20. Maheshri, N., Koerber, J.T., Kaspar, B.K., and Schaffer, D.V. (2006). Directed evolution of adeno-associated virus yields enhanced gene delivery vectors. Nat. Biotechnol. *24*, 198–204. https://doi.org/10.1038/nbt1182.

21. Huttner, N.A., Girod, A., Perabo, L., Edbauer, D., Kleinschmidt, J.A., Büning, H., and Hallek, M. (2003). Genetic modifications of the adeno-associated virus type 2 capsid reduce the affinity and the neutralizing effects of human serum antibodies. Gene Ther. *10*, 2139–2147. https://doi.org/10.1038/sj.gt.3302123.

22. Maersch, S., Huber, A., Büning, H., Hallek, M., and Perabo, L. (2010). Optimization of stealth adeno-associated virus vectors by randomization of immunogenic epitopes. Virology *397*, 167–175. https://doi.org/10.1016/j.virol.2009.10.021.

23. Avalos, A.M., and Ploegh, H.L. (2014). Early BCR Events and Antigen Capture, Processing, and Loading on MHC Class II on B Cells. Front. Immunol. *5*, 92. https://doi.org/10.3389/fimmu.2014.00092.

24. Forsstrom, B., Axnas, B.B., Rockberg, J., Danielsson, H., Bohlin, A., and Uhlen, M. (2015). Dissecting antibodies with regards to linear and conformational epitopes. PLoS One *10*, e0121673. https://doi.org/10.1371/journal.pone.0121673.

25. Jespersen, M.C., Peters, B., Nielsen, M., and Marcatili, P. (2017). BepiPred-2.0: improving sequence-based B-cell epitope prediction using conformational epitopes. Nucleic Acids Res. *45*, W24–W29. https://doi.org/10.1093/nar/gkx346.

26. Jin, X., Zhou, W., Luo, M., Wang, P., Xu, Z., Ma, K., Cao, H., Xu, C., Huang, Y., Cheng, R., et al. (2021). Global characterization of B cell receptor repertoire in COVID-19 patients by single-cell V(D)J sequencing. Brief. Bioinform. *22*, bbab192. https://doi.org/10.1093/bib/bbab192.

27. Raybould, M.I.J., Rees, A.R., and Deane, C.M. (2021). Current strategies for detecting functional convergence across B-cell receptor repertoires. mAbs *13*, 1996732. https://doi.org/10.1080/19420862.2021.1996732.

28. Pei, X., Earley, L.F., He, Y., Chen, X., Hall, N.E., Samulski, R.J., and Li, C. (2018). Efficient Capsid Antigen Presentation From Adeno-Associated Virus Empty Virions *In Vivo*. Front. Immunol. *9*, 844. https://doi.org/10.3389/fimmu.2018.00844.

29. Flotte, T.R. (2017). Empty Adeno-Associated Virus Capsids: Contaminant or Natural Decoy? Hum. Gene Ther. *28*, 147–148. https://doi.org/10.1089/hum.2017.29039.trf.

30. Xie, Y.L., Wang, J.Y., He, Y., Yu, X.M., Zheng, Q.Y., Ling, C., Feng, X.L., and Zhu, L.Q. (2023). The use of melittin to enhance transgene expression mediated by recombinant adeno-associated virus serotype 2 vectors both *in vitro* and *in vivo*. J. Integr. Med. *21*, 106–115. https://doi.org/10.1016/j.joim.2022.10.003.

31. Zheng, Q., Wang, T., Zhu, X., Tian, X., Zhong, C., Chang, G., Ran, G., Xie, Y., Zhao, B., Zhu, L., and Ling, C. (2021). Low endotoxin E. coli strain-derived plasmids reduce rAAV vector-mediated immune responses both *in vitro* and *in vivo*. Mol. Ther. Methods Clin. Dev. *22*, 293–303. https://doi.org/10.1016/j.omtm.2021.06.009.

32. Chai, Z., Zhang, X., Dobbins, A.L., Samulski, R.J., Merricks, E.P., Nichols, T.C., and Li, C. (2022). Dexamethasone Transiently Enhances Transgene Expression in the Liver When Administered at Late-Phase Post Long-Term Adeno-Associated Virus Transduction. Hum. Gene Ther. *33*, 119–130. https://doi.org/10.1089/hum.2021.083.

33. Chai, Z., Zhang, X., Dobbins, A.L., Rigsbee, K.M., Wang, B., Samulski, R.J., and Li, C. (2019). Optimization of Dexamethasone Administration for Maintaining Global Transduction Efficacy of Adeno-Associated Virus Serotype 9. Hum. Gene Ther. *30*, 829–840. https://doi.org/10.1089/hum.2018.233.

34. Trier, N.H., Hansen, P.R., and Houen, G. (2012). Production and characterization of peptide antibodies. Methods *56*, 136–144. https://doi.org/10.1016/j.ymeth.2011.12.001.

35. Xue, F., Li, H., Wu, X., Liu, W., Zhang, F., Tang, D., Chen, Y., Wang, W., Chi, Y., Zheng, J., et al. (2022). Safety and activity of an engineered, liver-tropic adeno-associated virus vector expressing a hyperactive Padua factor IX administered with prophylactic glucocorticoids in patients with haemophilia B: a single-centre, single-arm, phase 1, pilot trial. Lancet. Haematol. *9*, e504–e513. https://doi.org/10.1016/S2352-3026(22)00113-2.

OMTM, Volume *32*

Supplemental information

An investigation of the immune epitopes of adeno-associated virus capsid-derived peptides among hemophilia patients

Li Liu, Bingqi Xu, Lingling Chen, Jia Liu, Wei Liu, Feng Xue, Sizhou Feng, Erlie Jiang, Mingzhe Han, Wenwei Shao, Lei Zhang, and Xiaolei Pei

MAADGYLPDWLEDTLSEGIRQWWKLKPGPPPPKPAERHKDDSRGLVLPGYKYLGPFNGL
DKGEPVNEADAAALEHDKAYDRQLDSGDNPYLKYNHADAEFQERLKEDTSFGGNLGRA
VFQAKKRVLEPLGLVEEPVKTAPGKKRPVEHSPVEPDSSSGTGKAGQQPARKRLNFGQTG
DADSVPDPQPLGQPPAAPSGLGTNTMATGSGAPMADNNEGADGVGNSSGNWHCDSTW
MGDRVITTSTRTWALPTYNNHLYKQISSQSGASNDNHYFGYSTPWGYFDFNRFHCHFSPR
DWQRLINNNWGFRPKRLNFKLFNIQVKEVTQNDGTTTIANNLTSTVQVFTDSEYQLPYVL
GSAHQGCLPPFPADVFMVPQYGYLTLNNGSQAVGRSSFYCLEYFPSQMLRTGNNFTFSYT
FEDVPFHSSYAHSQSLDRLMNPLIDQYLYYLSRTNTPSGTTTQSRLQFSQAGASDIRDQSR
NWLPGPCYRQQRVSKTSADNNNSEYSWTGATKYHLNGRDSLVNPGPAMASHKDDEEKF
FPQSGVLIFGKQGSEKTNVDIEKVMITDEEEIRTTNPVATEQYGSVSTNLQRGNRQAATAD
VNTQGVLPGMVWQDRDVYLQGPIWAKIPHTDGHFHPSPLMGGFGLKHPPPQILIKNTPVP
ANPSTTFSAAKFASFITQYSTGQVSVEIEWELQKENSKRWNPEIQYTSNYNKSVNVDFTVD
TNGVYSEPRPIGTRYLTRNL

Figure S1. The respective positions of the peptides are highlighted.

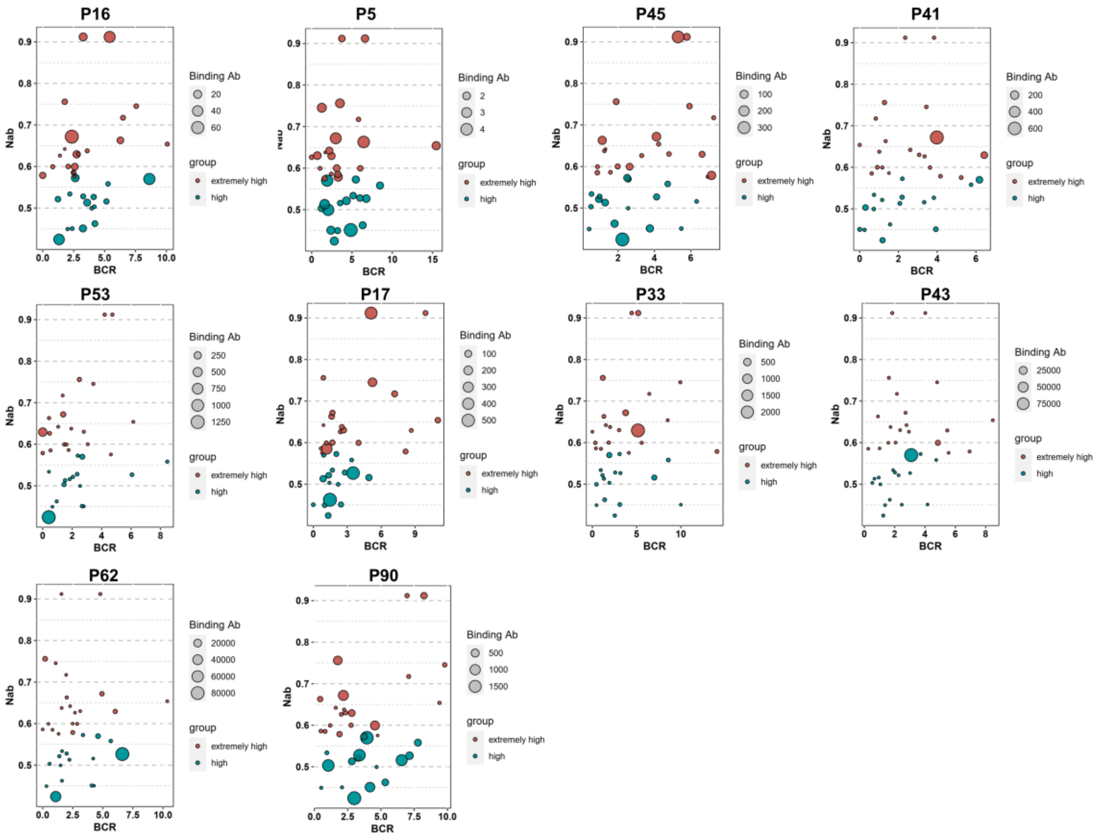

Figure S2. The epitopes of peptides, were assessed for Nab, Bab, and BCR recognition in both serum and PBMC samples obtained from hemophilia A/B patients with high or extremely high Nab titers. The calculated epitope profiles were presented.

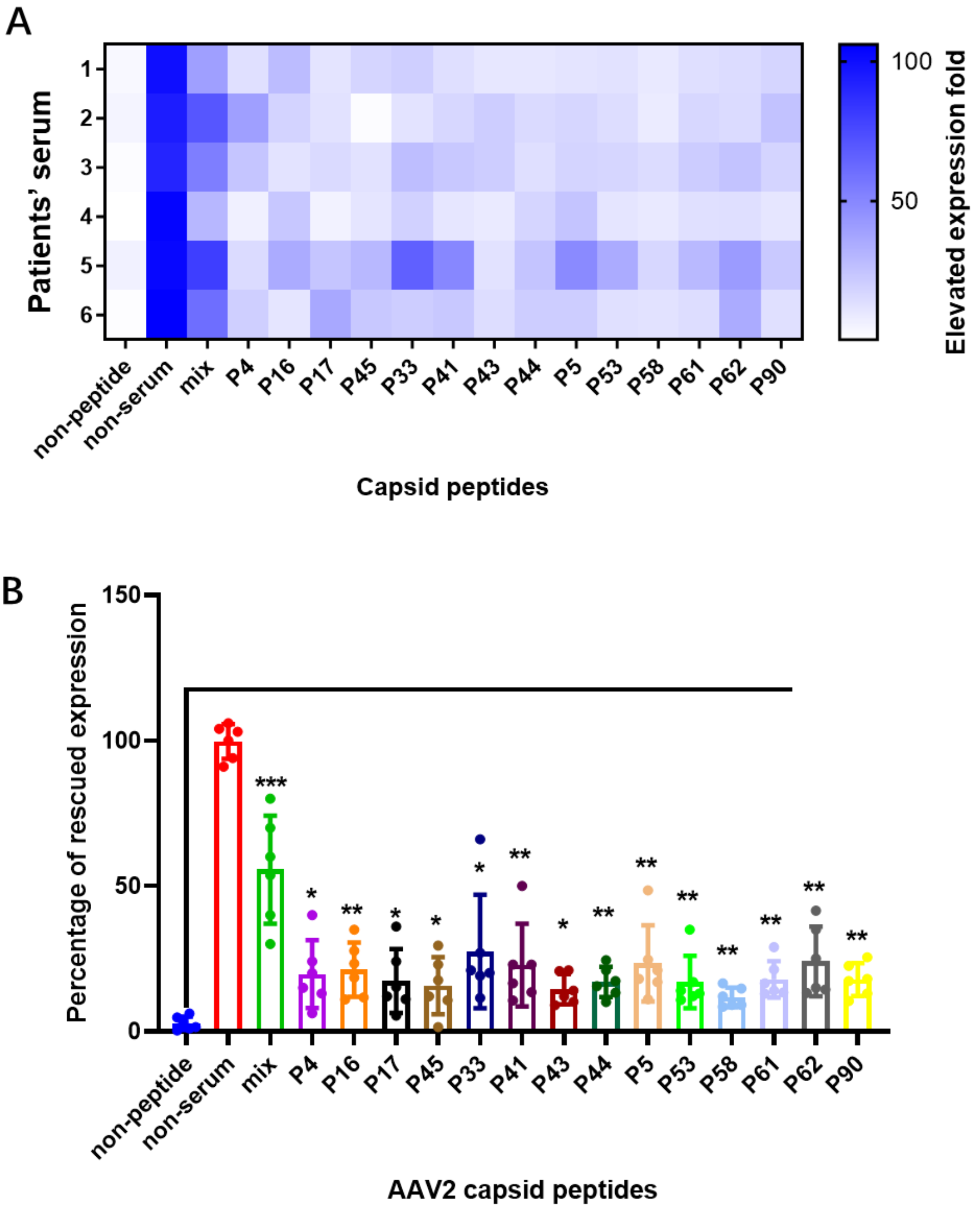

Figure S3. The inhibitory potential of epitope peptides was assessed using an in vitro experiment of AAV gene transduction to HEK293 cells. (**A&B**) In this model, compared with non-peptides-incubated group,the fold change of the rescued expression in luciferase activity was measured and analyzed to determine the extent of blocking achieved when serum (1 µL per well in 96-well plate) with the extremely high Nab titer to AAV2 (dilution > 1:128) was pretreated with single peptide (1 µg/mL) or a mixture

of epitope peptides (1 μg/mL), and then 1e8 vg AAV2-luc particles were added in the mixture, finally the serum-peptide-AAV2 mixture were added in the supernatant of HHEK293 cells.

Dilution	serum-1	serum-2	serum-3	serum-4	serum-5	serum-6	serum-7	serum-8	serum-9
1:1	354	669	765	351	250	558	226	355	655
1:2	563	859	946	691	774	767	441	618	783
1:4	1353	2816	2139	1945	720	2034	1004	2832	1525
1:8	4209	3643	5395	6390	4611	5331	2050	4540	8651
1:16	7820	8554	8257	7209	8846	13949	9231	8551	9767
1:32	17545	15984	19763	10260	9481	22427	18364	13409	13905
1:64	28731	28779	25332	26252	24221	29061	25721	19006	23643
1:128	46146	39128	32871	30789	27076	33025	32976	32079	28898
1:256	42445	42697	44300	43886	32868	40135	46999	40570	38370
1:512	54453	51438	44695	44629	40031	47774	50022	50321	47301
no-serum	52204	54662	51074	58745	50577	51637	53846	57007	56365

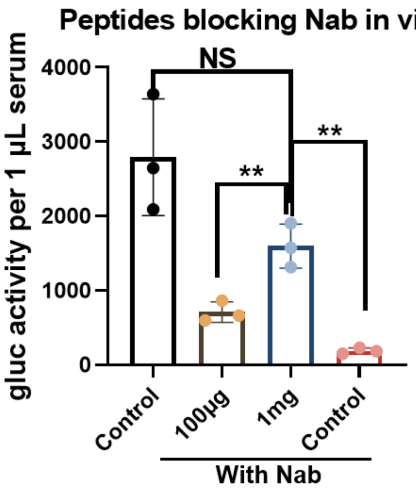

Figure S4. The female C57 mice, aged 6-8 weeks, were selected and retro-orbitally injected AAV2 virus particles (AAV2-GFP, 1e10 vg per mouse) into the mice. After one week, we collected serum from the mice and determined the titer of AAV2 neutralizing antibodies through in vitro neutralizing antibody assay. The neutralizing antibody titer against AAV2 in peripheral blood serum of the mice reached 1:64 (using the same Nab assay method as in the manuscript). Subsequently, we injected the entire peptide mixture (100μg or 1mg per mouse in 300 μl PBS) intravenously into the mice. One hour later, we intravenously injected 1e11 vg AAV2-Gluc (Gaussia Luciferase) per mouse, and on the 7th day, we collected venous blood from the mice to assess luciferase activity.

Table S1. The peptides were from the AAV2 capsid amino acid sequence that can recognized by the AAV2 neutralizing antibody, A20.

seq	length	id
DWLEDTLSEGIRQWWKLKPG	20	P4
EGIRQWWKLKPGPPPPKPAE	20	P5
KEDTSFGGNLGRAVFQAKKR	20	P16
NLGRAVFQAKKRVLEPLGLV	20	P17
ALPTYNNHLYKQISSQSGAS	20	P33
GFRPKRLNFKLFNIQVKEVT	20	P41
KEVTQNDGTTTIANNLTSTV	20	P43
TTTIANNLTSTVQVFTDSEY	20	P44
TSTVQVFTDSEYQLPYVLGS	20	P45
QMLRTGNNFTFSYTFEDVPF	20	P53
PLIDQYLYYLSRTNTPSGTT	20	P58
AGASDIRDQSRNWLPGPCYR	20	P61
CYRQQRVSKTSADNNNSEYS	20	P62
EIQYTSNYNKSVNVDFTVDT	20	P90
